# Myeloid sarcoma and pathological fracture: a case report and review of literature

**DOI:** 10.1007/s12185-023-03656-1

**Published:** 2023-09-14

**Authors:** Sho Takeyasu, Ken Morita, Seitaro Saito, Masanori Toho, Takashi Oyama, Takafumi Obo, Kazuki Taoka, Arika Shimura, Hiroaki Maki, Eisuke Shibata, Yusuke Watanabe, Fumio Suzuki, Liuzhe Zhang, Hiroshi Kobayashi, Munetoshi Hinata, Mineo Kurokawa

**Affiliations:** 1 Department of Hematology and Oncology, Graduate School of Medicine, The University of Tokyo, 7-3-1 Hongo, Bunkyo-Ku, Tokyo, 113-8655 Japan; 2 Department of Radiology, Graduate School of Medicine, The University of Tokyo, Tokyo, Japan; 3 Department of Orthopedic Surgery, Graduate School of Medicine, The University of Tokyo, Tokyo, Japan; 4 Department of Pathology, Graduate School of Medicine, The University of Tokyo, Tokyo, Japan

**Keywords:** Myeloid sarcoma, Pathological fracture, Myelodysplastic syndromes/neoplasms

## Abstract

Myeloid sarcoma is a rare clinical entity that presents as an isolated proliferation of leukemic cells, concurrently with or at relapse of acute myeloid leukemia (AML), myelodysplastic syndromes/neoplasms (MDS), chronic myeloid leukemia (CML), and myeloproliferative neoplasm (MPN). Myeloid sarcoma disrupts the normal architecture of its surrounding tissues. When it forms in long bones, it can cause their pathological fracture. We recently experienced a rare case of MDS presenting with myeloid sarcoma in the femur that eventually resulted in its pathological fracture. Detailed chromosomal analysis of the bone marrow cells suggested emergence of myeloid sarcoma during the fast-paced progression of MDS just after acquiring trisomy 22. A comprehensive review of previous cases of myeloid sarcoma-associated pathological fracture indicated possible involvement of structural rearrangements of chromosomes 9 and 22. Management of myeloid sarcoma should continue to improve, and clinicians should note that myeloid sarcoma with specific chromosomal alterations needs extra medical attention to prevent pathological fracture.

## Introduction

Myeloid sarcoma is a tumor mass consisting of myeloblasts with or without maturation involving any anatomical site. Most often, myeloid sarcoma is formed in patients with acute myeloid leukemia (AML), either at the time of diagnosis, during or after chemotherapy. This medical condition is well established in AML patients with recurrent genetic abnormalities, especially in cases of core binding factor AML that are characterized by the presence of either t(8;21) (q22;q22) or inv(16) (p13;q22) [1].

Among patients with myeloid sarcoma, pathological fracture is a rare yet highly important manifestation because of its tremendous impact on the patients’ quality of life. We have recently experienced a case of MDS presented with a myeloid sarcoma in the femur, which led to its pathological fracture. Besides presenting ours, in this report, we review previously reported cases of myeloid sarcoma-associated pathological fracture and discuss our findings with particular emphasis on the potential involvement of specific chromosomal aberrations in its pathogenesis.

## Case

**Fig. 1:**
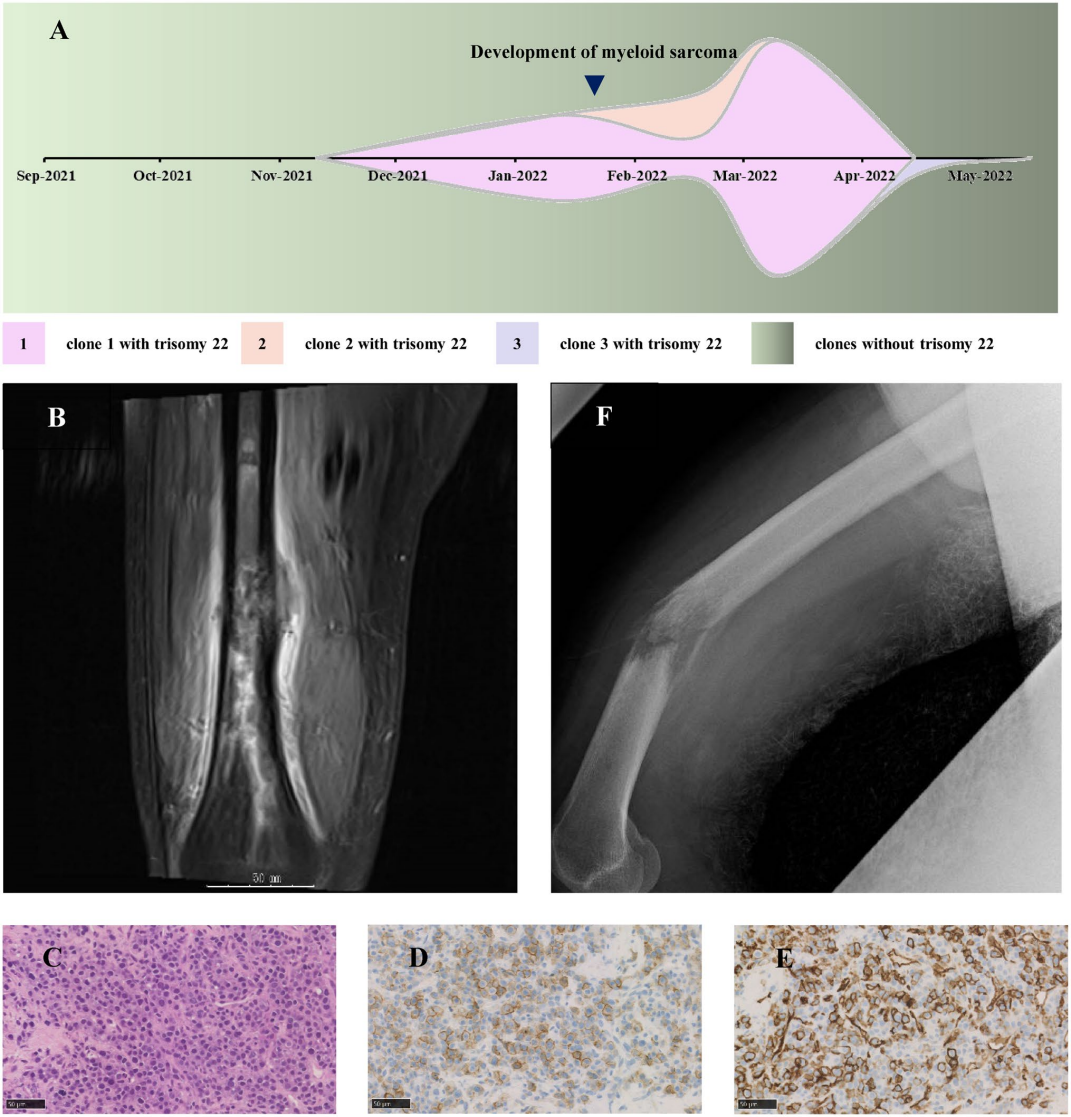
Myeloid sarcoma-associated pathological fracture. **A** Clonal evolution of MDS/AML cells with trisomy 22 in the bone marrow. Chromosomal analysis of the bone marrow cells showed additional chromosomal alterations including + 22 that had been observed just before the development of myeloid sarcoma in the right femur. (A-1) 45, XY, − 3, add (5)(q11.2), − 7, − 9, add(12)(p11.2), − 17, add(19)(p13.3), -20, + 22, + r, + 2mar, 2dmin. (A-2) 46, XY, add(5)(q11.2), − 7, + add(8)(q24.1), − 9, + 11, add(15)(p11.2), − 17, − 19, der(20)t(7;20), (q11.2;q13.1), + 22, + r, 2dmin. (A-3) 45, XY, − 1, add(1)(q21), − 3, add(5)(q11.2), − 7, − 9, − 12, − 17, add(19)(p13.3), − 20, + 22, + r, + 4mar, 3dmin. **B** Post-contrast fat-suppressed T1-weighted magnetic resonance image (MRI) of the myeloid sarcoma in the right femur presenting as an osteolytic lesion. **C**–**E** Histopathological analysis of the sample obtained from the fine needle biopsy of the myeloid sarcoma in the right femur. The Hematoxylin and Eosin (H & E)-staining revealed dense proliferation of immature myeloid cells with nuclear enlargement and high nuclear to cytoplasmic (N/C) ratio (**D**). The immature myeloid cells were stained positive for c-kit (**E**) and partially positive for CD34 (**F**). **F** Plain X-ray image of the myeloid sarcoma in the right femur presenting as a pathological fracture

In June 2021, a 59-year-old Japanese male with a history of lumbar compression fracture visited a local clinic complaining of fever lasting for five days. A systemic computed tomography (CT) scan was immediately taken, but did not locate any origins of fever. The complete blood counts showed white blood cells (WBC) of 6,700 /μL (13% neutrophils) and platelets of 1.02 × 10^5^ /μL, with normal hemoglobin levels of 11.4 g/dL. Detailed examination of the peripheral blood identified emergence of neutrophils with decreased cytoplasmic granules or with pseudo-Pelger-Huёt nuclear anomaly, and giant platelets. Analysis of the bone marrow aspiration showed hypercellular bone marrow with increased number of myeloblasts (4.6%) and ring sideroblasts (77%). Multilineage dysplasia of the hematopoietic cells were also evident, such as nuclear multilobation and nuclear budding erythroblasts, and megakaryocytes with separated nuclear lobes. The patient was thus diagnosed as MDS with low blasts and ring sideroblasts. In September 2021, the patient was referred to our hospital. The bone marrow examination was repeated, which detected increased number of myeloblasts to 7.8%. Cytogenetic analysis of the bone marrow showed complex karyotype: 45, XY, add(5) (q11.2), add(7) (p13), + 8, − 9, − 17, add(19) (p13.3), − 20, + r, 2 ~ 6dim [20/20] (Fig. 1A). The fluorescence in situ hybridization (FISH) resulted positive for probes detecting deletion of 5q, but negative for probes detecting monosomy 7. The patient decided to receive an allogenic hematopoietic stem cell transplantation (HSCT) as a curative therapy for MDS, intermediate-2 risk according to the International Prognostic Scoring System (IPSS), and we started coordination of bone marrow donors. In January 2022, the bone marrow aspiration was repeated to evaluate the progression of the disease. Chromosomal analysis showed complex karyotype containing + 22 that had not been recognized in September 2021: 40, X, -Y, − 5, add(7)(p13), − 9, add(15)(p11.2), -16, -17, -18, add(19)(p13.3), − 20, + r, 2dmin [8/20]/45, XY, − 3, add(5)(q11.2), − 7, − 9, add(12)(p11.2), − 17, add(19)(p13.3), − 20, + 22, + r, + 2mar, 2dmin [4/20]/44, XY, add(5)(q11.2), del(7)(p13), − 9, add(11)(p11.2), − 16, − 17, add(19)(p13.3), − 20, + r, + mar, 2dimn [3/20]/karyotypes similar to above (including add(5)(q11.2), add(19)(p13.3), and dmin) [5/20] (Fig. 1A-1). In February 2022, the patient was admitted to the hospital to receive a bridging chemotherapy for HSCT. At presentation, the patient complained of modest pain in the right thigh that started three weeks prior to admission. Because of persisting pain, a bone X-ray was taken, which showed presence of an osteolytic lesion in the right femur. Magnetic resonance imaging (MRI) showed a 45 mm mass in the right distal femur with moderate enhancement on post-contrast fat-suppressed T1-weighted images, which invades the tissue outside of the bone (Fig. 1B). Similar pathological lesions were also recognized in the right proximal femur and the right pelvis. The Hematoxylin and Eosin (H & E) stain of the fine needle-biopsied samples from the right femur showed proliferation of immature myeloid cells with atypical nucleus (Fig. 1C). The immunohistochemistry analysis of the fine needle-biopsied samples from the right femur showed infiltrating myeloblasts that were positive for vimentin, leukocyte common antigen (LCA), c-kit (Fig. 1D) and CD34 (Fig. 1E), while negative for cytokeratin AE1/AE3, S100, MPO (Myeloperoxidase), CD3 and CD20. These myeloblasts were morphologically and immunohistochemically considered identical to those in the bone marrow. The first course of azacitidine treatment was subsequently administered. After the diagnosis of the myeloid sarcoma, the weight bearing on the right leg was immediately restricted, and the patient was instructed to walk with double crutches. However, in March 2022, the patient complained of severe pain and swelling in the right thigh just after he twisted the right leg while changing body position on the bed. A plain X-ray picture was immediately taken, which showed pathological fracture of the right femur (Fig. 1F). The complete blood counts of the peripheral blood showed increased number of myeloblasts to 24.0%. Chromosomal analysis of the bone marrow cells revealed expansion of the clones that harbor trisomy 22 (Fig. 1A-2). With the diagnosis of MDS-overt AML, the patient underwent intensive chemotherapy with cytarabine and idarubicin. The pathological fracture of the right femur was managed conservatively without surgical intervention. This was because the surgical fixation of the fracture was considered to be at high risk for infectious complications, given the immunosuppression in uncontrolled AML. The induction chemotherapy with cytarabine and idarubicin achieved complete remission with incomplete count recovery (CRi). The patient then underwent consolidative chemotherapy with high-dose cytarabine in April 2022. The bone marrow examination in the next month, however, showed increased number of myeloblasts and proerythroblasts. Reinduction chemotherapy with azacitidine and venetoclax was initiated, yet was not effective enough to control the rapidly proliferating AML cells. The patient succumbed to death in June 2022 due to the progression of the disease.

## Discussion

Myeloid sarcoma is a tumor composed of immature myeloid precursor cells. It sometimes develops as a de novo isolated sarcoma without bone marrow involvement [2]. Besides AML (78.4%), it can be formed during the course of MDS, CML, and MPN [3, 4]. The myeloid sarcoma in the present case developed during the progression of the disease from MDS to AML. Myeloid sarcoma is not a common disease, and it is reported in 2.5–9.1% of patients with AML [5–7]. The most frequent chromosomal alterations are t(8;21) or inv(16) [1]. The skin, lymph node, testis, intestine, bone (3.3%) and central nervous system are the most frequently involved sites of myeloid sarcoma [8]. Notably, MDS cases with myeloid sarcoma have a poorer prognosis than those with isolated myeloid sarcoma or AML cases with myeloid sarcoma [9]. Besides t(8;21) and inv(16), the chromosomal alterations that had frequently been associated with myeloid sarcoma regardless of involved sites are t(9;11), tetrasomy 8, + 22, del(16q), + 4, + 8, t(8;17), t(8;16) [1].

As shown in Table 1**,** extensive review of the past literatures on myeloid sarcoma-associated pathological fractures, we have identified 18 cases that had been reported with description of the results of cytogenetic analyses. In a sharp contrast to the chromosomal alterations seen in myeloid sarcoma in general, our detailed review elucidated that the incidence of t(9;22) in myeloid sarcoma with pathological fractures (8/19 cases) is considered to be much higher than expected. Thus, we suspect the potential yet vital role of chromosomes 9 and/or 22 alterations in the development of myeloid sarcoma-associated pathological fractures. Given that the present case developed myeloid sarcoma soon after the immature myeloid cells acquired additional chromosomal alteration of + 22 (Fig. 1A-1), genes that are on chromosome 22 might be responsible for the pathological fracture with myeloid sarcoma. Intriguingly, chromosomal alteration of trisomy 22 had been identified in multiple cases of myeloid sarcoma at various sites [10–13]. In addition, although the role of chromosome 22 acquisition has not been fully elucidated in the pathogenesis of hematological malignancies, AML cases with inv(16) or t(16;16), which have higher chance to develop myeloid sarcoma, have been known to frequently be accompanied by trisomy 22 (24%) [14]. These findings stimulate the cancer biologists to study the role of genetic alterations involving chromosome 22 in the pathology of myeloid sarcoma and pathological fracture in future studies.

**Table 1:** Cytogenetic characteristics in previously reported cases of myeloid sarcoma-associated pathological fracture

Age	Sex	Etiology	Cytogenetic characteristics	Involved site	Treatment	Survival	Reference
59	M	MDS	45, XY, -3, add(5) (q11.2), -7, -9, add(12) (p11.2), -17, add(19) (p13.3), -20, + 22, + r, + 2mar, 2dmin [4/20] (Fig. 1A-1)	Femur	AraC + IDR High dose AraC Ven/Aza	Dead, 4 m	Our case
77	F	MDS	46, XX (del(5q) FISH +)	T8 spinal cord T7-8 vertebrae	Surgery	N/A	[15]
19	M	Isolated sarcoma	t(9;22) (q34;q11.2)	Humerus	Intensive chemotherapy Allo-PBSCT Local radiotherapy	Alive, 2 y after SCT	[16]
61	M	Isolated sarcoma	t(9;22) (q34;q11.2)	Femur	Chemotherapy Imatinib	Alive, 1 y 10 m	[17]
82	F	Isolated sarcoma	46, XX	Humurus	Local radiotherapy	Alive, 9.5 y	[18]
69	F	Isolated sarcoma	46, XX	Humurus	CHOP AraC + IDR Local radiotherapy	Alive, 2 y 5 m	[19]
82	M	Isolated sarcoma	46, XY, t(15;17)	11^th^ rib	ATRA + IDR ATRA + Arsenic trioxide	Alive, 11 m	[20]
56	M	AML	Complex karyotype including deletion of 5q, trisomy 8, and additional material on chr. 17	Femur	Imatinib Surgery	Dead, 5 d after surgery	[21]
64	M	AML	46, XY 45, X, -Y	10^th^ rib	Low dose AraC MZT + High dose AraC AraC + Thioguanine + DNR	Alive, 1 y	[22]
0	M	AML	Trisomy 10	Orbital wall	Chemotherapy	Alive	[23]
18	M	CML	t(9;22)	Humerus	Nilotinib	Alive, 2 y	[24]
49	M	CML	t(9;22)	Femur	Busulfan Local radiotherapy Surgery	N/A	[25]
3	F	CML	Complex karyotype including t(9;22) (q34;q11)	Femur	Imatinib + HU + 6MP Surgery AraC AraC + IDR Local radiotherapy	Dead, 4 m	[26]
39	M	CML	t(9;22)	Humerus	Imatinib AraC + High dose DEX Local radiotherapy	Dead, 2 m	[27]
62	M	CML	64, XY with complex karyotype including t(9;22)	L2 vertebra	Imatinib	Dead	[28]
50	F	CML	t(9;22)	Femur	Surgery radiotherapy	N/A	[29]
59	F	ET	46, XX, + 1, der (1;13) (q10;q10), del(7) (q22q32)	Humerus	Imatinib AraC + ETP	Dead, 1 m	[30]
50	M	MPN	46, XY (BCR-ABL1 FISH +)	Femur	Dasatinib + AraC SCT	Dead, 76 d after SCT	[31]
63	M	MF	JAK2 +	Femur	Ruxolitinib	Dead, 10 m	[32]

*MDS* Myelodysplastic syndromes/neoplasms, *AML* Acute myeloid leukemia, *CML* Chronic myeloid leukemia, *ET* Essential thrombocythemia, *MPN* Myeloproliferative neoplasms, *MF* Myelofibrosis, *chr.* chromosome, *BCR-ABL1* Breakpoint cluster region-Abelson 1, *FISH* Fluorescence in situ hybridization, JA*K*2 Janaus kinase 2, *AraC* Cytarabin, *IDR* Idarubicin, *Ven* Venetoclax, *Aza* Azacitidine, *Allo-PBSCT* Allogeneic peripheral blood stem cell transplantation, *CHOP* Cyclophosphamide, Doxorubicin, Vincristine, and Prednisolone, *ATRA* All trans-retinoic acid, *MZT* Mitoxantrone, *DNR* Daunorubicin, *HU* Hydroxyurea, *6MP* 6-mercaptopurine, *DEX* Dexamethasone, *ETP* Etoposide, *SCT* Stem cell transplantation.

In summary, we have experienced a rare case of MDS presented with a myeloid sarcoma in the right femur that eventually resulted in its pathological fracture. Reviewing the past cases, the possible involvement of the rearrangements of chromosomes 9 and 22 is suspected in the occurrence of myeloid sarcoma-associated pathological fractures. To seek the optimal management of myeloid sarcomas and prevention of associated complications, clinicians are encouraged to pay extra medical attention to the myeloid sarcoma cases involving chromosomes 9 or 22 alterations.

## Data Availability

Relevant clinical data is available upon request.
